# A New Implantable Closed-Loop Clinical Neural Interface: First Application in Parkinson’s Disease

**DOI:** 10.3389/fnins.2021.763235

**Published:** 2021-12-07

**Authors:** Mattia Arlotti, Matteo Colombo, Andrea Bonfanti, Tomasz Mandat, Michele Maria Lanotte, Elena Pirola, Linda Borellini, Paolo Rampini, Roberto Eleopra, Sara Rinaldo, Luigi Romito, Marcus L. F. Janssen, Alberto Priori, Sara Marceglia

**Affiliations:** ^1^Newronika SpA, Milan, Italy; ^2^Dipartimento di Elettronica, Informazione e Bioingegneria, Politecnico di Milano, Milan, Italy; ^3^Narodowy Instytut Onkologii im. Marii Skłodowskiej-Curie, Warsaw, Poland; ^4^Department of Neuroscience, University of Torino, Torino, Italy; ^5^AOU Città della Salute e della Scienza, Molinette Hospital, Turin, Italy; ^6^Fondazione IRCCS Ca’ Granda Ospedale Maggiore Policlinico, Milan, Italy; ^7^Movement Disorders Unit, Department of Clinical Neurosciences, Fondazione IRCCS Istituto Neurologico C. Besta, Milan, Italy; ^8^Department of Neurology and Clinical Neurophysiology, Maastricht University Medical Center, Maastricht, Netherlands; ^9^Faculty of Health, Medicine and Life Sciences, School for Mental Health and Neuroscience, Maastricht University, Maastricht, Netherlands; ^10^Department of Health Sciences, Aldo Ravelli Research Center for Neurotechnology and Experimental Neurotherapeutics, University of Milan, Milan, Italy; ^11^Dipartimento di Ingegneria e Architettura, Università degli Studi di Trieste, Trieste, Italy

**Keywords:** deep brain stimulation, neuromodulation, closed-loop, local field potential (LFP), Parkinson’s disease, neural interface, implantable device

## Abstract

Deep brain stimulation (DBS) is used for the treatment of movement disorders, including Parkinson’s disease, dystonia, and essential tremor, and has shown clinical benefits in other brain disorders. A natural path for the improvement of this technique is to continuously observe the stimulation effects on patient symptoms and neurophysiological markers. This requires the evolution of conventional deep brain stimulators to bidirectional interfaces, able to record, process, store, and wirelessly communicate neural signals in a robust and reliable fashion. Here, we present the architecture, design, and first use of an implantable stimulation and sensing interface (AlphaDBS^R^ System) characterized by artifact-free recording and distributed data management protocols. Its application in three patients with Parkinson’s disease (clinical trial n. NCT04681534) is shown as a proof of functioning of a clinically viable implanted brain-computer interface (BCI) for adaptive DBS. Reliable artifact free-recordings, and chronic long-term data and neural signal management are in place.

## Introduction

Deep brain stimulation (DBS) device and implant design was developed on the learnings and advancements owned by cardiac pacemakers. After the first commercially DBS device approved by Food and Drug Administration for Parkinson’s disease (PD) in 1997 (Paff et al., 2020), DBS technology did not witness significant advances, until recently, when new companies introduced technology innovations while entering the DBS market (Guidetti et al., 2021; Krauss et al., 2021). They include novel electrode designs and materials, stimulation waveforms, neural sensing capabilities, stimulation directionality, and battery size reduction with life extension (Krauss et al., 2021).

In particular, neural sensing is of critical importance to explore the pathophysiology of diseases targeted by DBS and, in turn, to develop new closed-loop devices (Starr, 2018; Gilron et al., 2021). In addition, a fully implantable device capable of bidirectional communication with the brain can be considered as a real brain-computer interface (BCI) implementation (Starr, 2018).

Signals recorded from DBS electrodes were used to gain insights into basal ganglia functioning both during intra-operative recording sessions and during peri-operative experimental settings [after the implant of the DBS electrode and before the connection of the implantable pulse generator (IPG)]. More specifically, local field potentials (LFPs), representing the compound activity of neuronal ensembles around DBS macroelectrode, are explored as a valuable feedback variable for closed-loop or adaptive DBS (aDBS) (Priori et al., 2013; Habets et al., 2018; Starr, 2018; Guidetti et al., 2021; Krauss et al., 2021).

In PD, oscillatory activity obtained by LFP recordings correlates with a range of symptomatic states (Brown, 2003; Priori et al., 2013; Arlotti et al., 2016a; Meidahl et al., 2017). These LFPs can be chronically recorded (Giannicola et al., 2012) and are modulated by DBS (Rossi et al., 2008; Giannicola et al., 2010; Eusebio et al., 2012). aDBS is coming closer to the clinical practice by increasing amount of proof of concept studies (Little et al., 2013, 2016a,b; Rosa et al., 2015, 2017; Piña-Fuentes et al., 2017; Arlotti et al., 2018; Swann et al., 2018; Velisar et al., 2019). LFPs have been proposed as a control variable for other pathologies including dystonia (Piña-Fuentes et al., 2020; Johnson et al., 2021), essential tremor (He et al., 2020; Opri et al., 2020), depressive and obsessive compulsive disorders (Neumann et al., 2014), and Tourette syndrome (Marceglia et al., 2017; Molina et al., 2017).

The first commercially available implantable neurostimulators with sensing capabilities was introduced for the treatment of epilepsy (Morrell and On behalf of the RNS System in Epilepsy Study Group, 2011). This device, which was then used for aDBS in Tourette syndrome (Molina et al., 2017), is able to record and analyze brain activity to provide a closed-loop stimulation. The aDBS strategy implemented follows the concept of “responsive neuromodulation” where stimulation is triggered on a determined event/episodes rather than being continuously administered. Although the paradigm of responsiveness is suitable for epilepsy or other disorders characterized by symptomatic episodes (i.e., Tourette), clinical applications as PD require continuous stimulation and simultaneous monitoring of the pathophysiological clinical state. The implementation of this type of devices, allowing continuous recording while stimulation is ON, faces a major challenge: recording signals having < 1 uV amplitude in occurrence of > 1 V stimulation artifact (Arlotti et al., 2016b; Zhou et al., 2019). Embedding concurrent sensing and stimulation circuitry in an implantable device is further complicated by the power and size constrains.

Here, we present an implantable neurostimulator for LFPs-based aDBS (AlphaDBS^R^ System), where the sensing problem is fully addressed. We discuss preliminary results with regard to the stimulation and sensing performances as tested in three patients with PD during a pilot study (clinical trial n. NCT04681534).

## State of the Art and Innovative Requirements

### Stimulation Design Inputs

In commercial systems for DBS treatment, stimulation parameters range from 0 to 25 mA of amplitude, from 10 to 450 μs of pulse-width, and 2 to 500 Hz of frequency, provided both in monopolar and bipolar fashion (Paff et al., 2020). Empirical observations showed that, in PD, clinical benefits can be fully achieved with a narrower parameters space. For instance, when considering patients with PD, tremor, bradykinesia, and rigidity progressively improved between 2 and 3 V and did not continue to improve beyond 3 V (Moro et al., 2002) that, for an average monopolar impedance of 1,000 Ω, is equal to 3 mA. In the absence of lead damages, for platinum-iridium electrode with area of 0.06 cm^2^, impedances may vary between 500 and 2 KΩ (Kuncel and Grill, 2004). In clinical practice, the amplitude threshold for inducing a clinical response or side effect for each electrode contact is determined by using monopolar stimulation and a stepwise increase in amplitude of 0.2–0.5 V (0.2–0.5 mA) (Volkmann et al., 2002), thus requiring a minimum amplitude resolution of 0.2 mA. For STN stimulation, a 60-μs pulse width is generally used because of its neurons’ chronaxie, and it was empirically observed as being effective on rigidity and bradykinesia (Moro et al., 2002). Lowering the pulse width helps in augmenting the therapeutic window based on the intensity-pulse duration chronaxie relationship (Reich et al., 2015). Frequency stimulation above 200 Hz (Moro et al., 2002) did not show any notable improvements, whereas frequencies below 50 Hz generally worsen Parkinsonian symptoms (Wojtecki et al., 2006).

Despite specific parameters choice, because the electrical safety of the stimulation has to be guaranteed, intrinsic constrains depend on the material and geometries of the electrodes. Stimulation waveforms shall be charge balanced, in active/or passive manner for preventing electrode and tissue interface damage (Cogan et al., 2004). Moreover, intensity and pulse width combination shall be controlled, on the basis of electrode surface, to avoid excessive charge density injection per phase (Merrill et al., 2005). In particular, for conventional platinum-iridium electrodes (i.e., Model 3389, Medtronic), the limit for charge density is 30 μC/cm^2^/phase.

In AlphaDBS System, the narrower parameter space (frequency of 50–200 Hz, pulse width of 40–250 μV, and amplitude of 0–5 mA), with charge balanced waveforms and with specific controls allowing to reliably guarantee electrical safety, is considered as stimulation requirement for AlphaDBS, without introducing any specific innovation in the stimulation module, which is an established technology. The parameters space, however, could be suitable also for other potential DBS applications (e.g., dystonia or tremor). The sensing and data management modules are the places where innovation for bidirectional neural interfaces is needed.

### Sensing Design Inputs

To implement aDBS, clinical IPGs need to record artifact-free neural activity during stimulation delivery.

**FIGURE 1:**
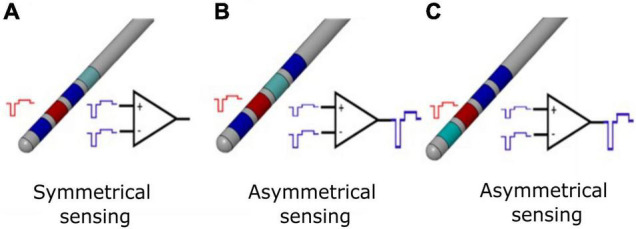
Differential sensing configurations for conventional DBS electrode. **(A)** Symmetrical sensing employs two recording contacts (blue) adjacent to the stimulation contact (red); at the inputs of the differential amplifier, the common mode stimulation artifact (in the ideal case of balanced impedances) is the same, and for an ideal common mode rejection ratio (CMMR), the output of the stimulation artifact is canceled by subtraction. **(B)** Asymmetrical sensing employs two recording contacts (blue) in the opposite position but at different distance compared to the stimulation contact (red), or two recording contacts (blue) in the same position and at different distance compared to the stimulation contact (red). At the inputs of the differential amplifier, the common mode stimulation artifacts (in the ideal case of balanced impedances) are not the same; even for an ideal CMMR, the output of the stimulation artifact is not canceled by subtraction. In real case scenario, impedances are unbalanced and the CMMR is not ideal; therefore, asymmetrical sensing implies a further worsening of the recording configuration. **(C)** Asymmetrical sensing with two adjacent contacts. The panel is organized as in (B) and the same comments apply.

Although external systems were able to solve the artifact rejection problem (Rossi et al., 2007; Arlotti et al., 2016b; Petkos et al., 2019), the size and power constrains of implantable operations make the rejection of stimulation artifact a technical implementation challenge. The stimulation artifact consists of direct components (stimulus time-locked voltage transients) and indirect components (voltage decay in the inter-pulse period) (Zhou et al., 2019). Direct artifacts at the adjacent recording electrodes are in the order of volts (common mode artifact) or hundreds of millivolts (differential mode artifact). In DBS applications, the differential artifact amplitude imposes a minimum input range of 100 mVpp, but real-world impedances mismatch may lead to greater values. The sensing module should avoid saturation for differential artifact greater than 100 mVpp while resolving 1-μV signals. In fact, as reported in the literature, implantable DBS devices with sensing capabilities (i.e., Medtronic Activa PC + S) are not able to provide artifact-free meaningful recordings (Cummins et al., 2021). Symmetric electrode configuration (Figure 1A) with input blanking has been applied as means to mitigate the differential and common mode artifacts (Stanslaski et al., 2018), providing better artifact management (Cummins et al., 2021) but introducing a limitation in choosing the best stimulation configuration for the patient.

Any combinations of electrode contacts should be selectable for recording with respect to the stimulation one (Figure 1). In the absence of this requirement, given a conventional quadripolar linear DBS lead, the selection of the extreme contacts would deny recording possibilities, because of the unavailability of recording contacts symmetrical to the stimulation one. Even worse, for directional leads (Eleopra et al., 2019), the electrode position together with the different area of directional contacts vs. cylindric contacts would lead both to unbalanced impedances and spatially asymmetrical recording contacts, thus deteriorating artifact rejection.

In case of clinical closed-loop DBS, a stimulation agnostic sensing module, able to reject the stimulation artifact independently from the stimulation shape and configuration (monopolar and bipolar), is preferrable to freely set the most effective stimulation. The possibility to be stimulation agnostic depends on the artifact rejection strategy. For instance, employing input blanking techniques, as described in Stanslaski et al. (2018), limits the choice of the stimulus shape. Ideally, the pulse waveform should be actively charge balanced and symmetrical to minimize the time duration of the stimulus artifact and maximize the benefit of blanking. Conversely, in monophasic passively charge-balanced stimulation, the voltage decay of a single pulse may last for hundreds of microseconds, thus requiring to increase the duration of blanking and data loss.

An optimal sensing module should be also software needless, not requiring for additional software for artifact mitigation or removal. Back-end software solutions have been proposed and implemented in other devices ranging from interpolation (Zhou et al., 2019), support vector machines (Stanslaski et al., 2018), and template matching (Qian et al., 2017). Real-time processing on implantable devices requires computational power, which, as a rule of thumb, should be minimized, but as long as back-end solutions prove to be compatible with low-power real-time processing constrains, they can still be employed.

Therefore, the requirements of the AlphaDBS sensing module to implement a bidirectional deep brain neurostimulator are (1) resolving 1 μV LFPs signals, (2) eliminating differential stimulus artifact (>100 mVpp) and common mode stimulus artifact (>1V), (3) being stimulation agnostic, (4) being electrode configuration independent, and (5) being needless for back-end processing. Requirements (3), (4), and (5) are innovative with respect to other available options of implantable DBS devices with sensing capabilities, altogether providing a reliable and robust system for artifact-free recordings.

### Data Management Design Inputs

Having the object of accelerating neurophysiological research, a core requirement for a bidirectional IPG acting as a clinical BCI is to store and transmit neural signals. Although chronic data streaming represents a heuristic goal, its practical implementation still needs to overcome important limitations such as high-power demand, consequent fast battery drain, and maintenance of a permanent external receiver link; all these features ultimately add unnecessary burdens for patients. For instance, continuous data streaming with an implantable rechargeable device (Gilron et al., 2021) require the use of a transmitter that has to be continuously worn by the patient. Many bidirectional neuromodulation platforms are targeting chronic wireless communication (Zhou et al., 2019) at the preclinical or investigational stage.

A less power-consuming solution for chronic neural activity monitoring is to collect data in an embedded memory located inside the IPG. Its implementation requires to compress the neural data in their spectral features or any features being relevant under a clinical and neurophysiological perspective. The correct trade-off between the tracking needs and the size of the embedded memory is application specific. For instance, in PD, the beta power time course is linked to daily motor fluctuations of the patients (Arlotti et al., 2018; Gilron et al., 2021), thus suggesting that extracting the beta power band and continuously storing it could provide an efficient clinical monitoring. However, available devices have limited memories that are overwritten if data are not downloaded and therefore have time-limited monitoring capabilities (Jimenez-Shahed, 2021).

Embedding compressed data (i.e., spectral power) requires to have an *a priori* knowledge of what signal features are significant for the specific disease, but because of the exploratory application of clinical BCI, time domain data are necessary to the discovery of new biomarkers and physiological mechanisms of action. Moving from the concept of chronic monitoring to exploratory recording, the requirement of data wireless streaming can be relaxed by limiting it to on-demand and time-constrained streaming sessions that allow for controlled experimental investigations without burdening the patient.

The AlphaDBS System will therefore implement two innovative features: (1) the continuous real-time streaming and visualization of data to be used in experimental settings and (2) a long-term continuous recording of embedded data, with an innovative download strategy guaranteeing no data loss.

### Processing Design Inputs

Adapting stimulation in real time requires to process a physiological variable and to calculate a new set of parameters based on a given relationship (proportional/adaptive mode) or a lookup table (digital mode or state machine). In the AlphaDBS System, the chosen requirement is to implement embedded data processing that ensures lower power consumption, better data privacy, and shorter time delays in stimulation changes, compared with external processing that, however, increases flexibility and research applicability (Pulliam et al., 2020).

Therefore, the AlphaDBS System is a fully closed-loop system, with an embedded algorithm that uses recorded LFPs as biomarker and adapts the stimulation amplitude accordingly, without the need of any external processing.

## Materials and Methods

### AlphaDBS System Architecture

**FIGURE 2:**
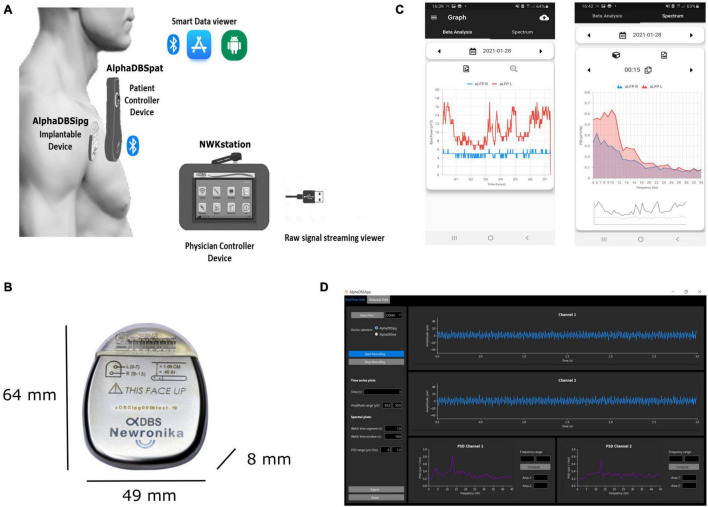
AlphaDBS System architecture. **(A)** AlphaDBS System components: the AlphaDBSipg implantable device is recharged using a patient controller (AlphaDBSpat) that also allows downloading data and signals recorded using the embedded mode. A mobile app allows data visualization. The physician controller device (NWKstation) is used to program the AlphaDBSipg and to visualize LFPs recorded in the streaming mode. **(B)** AlphaDBSipg dimensions. **(C)** Screenshot of the mobile app showing beta band amplitude time changes (on the left) and power spectrum at a given time point (on the right). **(D)** Python-based GUI for real-time LFPs processing, visualization, and storing.

According to the requirement defined above, the AlphaDBS system (Figure 2) consists of four main components: an IPG (AlphaDBSipg), a patient controller (AlphaDBSPat), a physician controller (NWKStation), and an external device for data recording and streaming from externalized leads (AlphaDBSext). These components together implement a distributed data management platform for data recording, processing, streaming, and storing.

The AlphaDBSipg sensing is implemented in two modes: the “embedded” mode and the “streaming” mode. In the embedded mode, the IPG records and stores neural data during chronic treatment delivery (conventional DBS, cDBS, or aDBS) in an embedded not volatile memory. During stimulation (either cDBS or aDBS), the system extracts the power value of a selected frequency band and stores one value for each side every minute, two full spectra (from 5 to 35 Hz, one per side) every 10 min, and two values of the stimulation amplitude every 10 min. The patient controller downloads the data stored in the embedded memory of the IPG at every recharging cycle and stores them in a second not volatile memory having higher capacity. These data can be downloaded to a smartphone or laptop through a Bluetooth connection using dedicated custom application programming interfaces (APIs). At present, embedded data downloaded through the AlphaDBSpat are transferred to an app that implements a fast healthcare interoperability resource (FHIR)-based standard data management (ready for future interoperability) and allows historical data visualization and power spectral analysis (Figure 2B). In the streaming mode, the IPG, on demand, streams data to the physician programmer (NWKStation) that acts as a receiver and, in turn, transmits data *via* UART-to-USB connection to a smartphone or a laptop, which can be used for data storing and visualization thanks to custom APIs. Figure 2C shows the present implementation of a Python-based graphic user interface (GUI) that receives, visualizes, and saves real-time data.

The AlphaDBSipg has a total volume of 20.96 cc and weight of 32.70 g, with a medical grade rechargeable battery of 200 mA/h, retaining the 90% of the capacity at 2,000 cycles. The size is in line with other rechargeable DBS IPGs, such as Boston Scientific Vercise (volume of 20.7 cc and weight of 33 g) and Medtronic Activa RC (volume of 22 cc and weight of 40 g). The header is compatible with Medtronic DBS lead extensions model 37086, and it can allocate two extensions for a total of 16 independent contacts. The AlphaDBSipg electronic board has circuitry for driving 16 stimulation channels, each of them can be configured independently. The output current for each channel ranges from 0 to 5 mA. Multisite stimulation is possible by keeping the duration and the frequency of the pulses fixed. The stimulation waveform is firmware selectable, with both active and passive charge balancing available. In case of active return, the ratio between the cathode and the anode current amplitude is 5, leading to a balancing anodic pulse lasting five times the cathodic one. The AlphaDBSipg has been configured for providing capacitive coupled active charge balanced asymmetric pulses, with frequency ranging between 40 and 200 Hz and pulse width ranging between 40 and 250 μs, despite that frequency can be extended to 2.5 KHz and pulse width to 1 ms. At 5 mA and 250 μs, the charge density injected per phase is 20 μV/cm^2^/phase, when considering a platinum-iridium electrode having a surface of 0.06 cm^2^ (i.e., Model 3389, Medtronic, Inc.) and the maximum charge density injection accepted is 30 μC/cm^2^/phase.

The AlphaDBSipg can deliver DBS both in the conventional mode (cDBS), in which stimulation parameters are set using the physician controller and remain fixed, and the adaptive mode (aDBS). In this last case, stimulation amplitude, pulse width, and frequency are dynamically changed on the basis of the embedded closed-loop logic or pre-set *via* physician programmer and radio frequency (RF) communication. The closed-loop logic implementation now tested is based on the linear proportional feedback mode that uses the LFP beta band (10–35 Hz) as neurophysiological biomarker (Arlotti et al., 2018; Guidetti et al., 2021). In summary, the specific personalized beta band of the patient is chosen by inspecting recorded LFPs using the streaming mode. Then, both the personalized beta band and the therapeutic window are set in the physician controller. When aDBS is ON, the recorded beta band is analyzed and the DBS amplitude is modulated linearly between the maximum and minimum amplitudes set as therapeutic window (Arlotti et al., 2018; Prenassi et al., 2021). The closed-loop logic is fully embedded and implemented at the microcontroller firmware level and does need for external units.

The inputs of two differential sensing channels can be multiplexed, respectively, on any of the eight contacts of each lead, and no blanking technique is used for artifact mitigation. The neural signals, analogically filtered for artifact suppression, are digitally converted by the analogue to digital converter (ADC) at a frequency of 512 Hz. Residual harmonic artifacts, when no completely suppressed, require for a sample frequency being greater than the double of the stimulation frequency to avoid aliasing. No additional digital signal processing for artifact removal is needed. The firmware is fully employed for feature extraction and closed-loop logic implementation. This patented sensing technology was (Priori et al., 2005) already implemented and illustrated in external devices (Rossi et al., 2007; Arlotti et al., 2016b) that were used to collect preliminary data on aDBS in more than 40 patients (Rosa et al., 2015, 2017; Arlotti et al., 2018, 2019; Bocci et al., 2021; Prenassi et al., 2021).

A 2.4-GHz ISM/SRD chip allows data streaming of two sensed signals for a distance up to 10 m, firmware upgrade, and bidirectional communication with the patient and the physician controller. The firmware is upgradable through an on-air boot loading functionality.

The AlphaDBSipg has two different microcontrollers: one dedicated to the sensing module, and one to manage the stimulation module, the battery functions, and the RF streaming.

The AlphaDBS System received european (CE) mark for conventional DBS and sensing in January 2021.

### System Use

#### Study Protocol and Surgery

The AlphaDBS system is undergoing clinical testing in a pilot multicenter randomized cross-over study on adaptive versus conventional DBS (aDBS vs. cDBS). All the details of the protocol are available on clinicaltrials.gov (study ID: NCT04681534). The study was approved by all regulatory authorities involved, and all the patients gave their informed consent to the study.

In summary, the study protocol is organized in two phases: the “short-term follow-up” (3 days in the hospital setting, 1 day for the system calibration + 1 day per each mode) and the “long-term follow-up” (1 month at home, 2 weeks per each mode). Patients with PD are screened from a population in need for IPG replacement for battery depletion if bilaterally treated using a Medtronic Activa PC or Activa RC IPG (mono-channel or dual channel) with DBS leads implanted in the STN (Model 3389) and extensions (Model 37806) compatible with the IPG of the AlphaDBS System (called AlphaDBSipg). The aDBS algorithm tested in this pilot study is the one reported in previous studies with external systems (Rosa et al., 2017; Arlotti et al., 2018).

During surgery for IPG replacement, after removal of the previously implanted device, *via* subclavicular incision, the DBS lead extensions were connected with a sterilized trial cable and an adapter to the external wireless recording device (AlphaDBSext) to test leads impedances and the presence of electrocardiographic artifact. After this check, the AlphaDBSipg was connected to Medtronic extensions with the patient under local anesthesia. In case of bilateral stimulation with two devices, the left DBS lead extension was replaced and transferred to the right side under general anesthesia to allow the replacement with a single IPG.

After IPG replacement, the impedances of each contact were measured again to ensure the absence of short/open circuits and also to confirm the consistency with the measurements done with the previous implant. Then, the new IPG was switched ON in continuous DBS (cDBS) with stimulation parameters selected in accordance with previous settings. In case of a previous voltage-controlled IPG, a simple translation to current on the basis of measured impedances parameter was performed following the Ohm’s law. The response of the patient was clinically assessed (Unified Parkinson’s Disease Rating Scale – part III in MedOFF/StimOFF and MedOFF/StimON) to further adjust stimulation parameters if needed.

Then, on days 2 and 3, patients entered the study protocol and underwent 2 days of stimulation, one in aDBS and one in cDBS (randomized), before being sent home for one additional month (2 weeks in aDBS and 2 weeks in cDBS, in the same order as during the short-term follow up).

Because the pilot study is still ongoing, here, we report only the results of neurophysiological recordings obtained from the first three patients enrolled. Clinical data cannot be reported until the end of the study.

#### In-Clinic Local Field Potential Data Collection

During hospitalization (short-term follow up), LFPs were recorded both in the streaming and in the embedded mode.

LFP streaming, as indicated in the design input, was limited to a short time window, whereas LFP recording using internal memory was always ON.

LFP streaming was used to choose the best contact pair to be used in chronic recording (calibration session on day 1). More specifically, LFPs were recorded and streamed out from all the possible contacts pairs (excluding the one used for stimulation) that, considering bilateral monopolar configuration, it includes six differential traces, three per side. The patients were in the MedOFF/StimOFF condition and were asked to stay in rest position during the data streaming. Each recording lasted 10 s, to minimize the time in which the patients experienced the return of motor symptoms (MedOFF/StimOFF condition). The power spectra were directly visualized for each trace, and the contact pair showing higher beta (10–35 Hz) activity was selected for chronic recording during DBS treatment. The recorded beta band is defined as ± 5 Hz from the peak frequency in the beta band (personalized beta band).

At the end of the experimental session with LFP data streaming, LFP chronic recording (embedded mode) was activated and consisted in storing physiological data inside the IPG on a not-volatile memory for chronic recording and offline downloading and processing. Embedded mode was switched ON continuously (except during LFP real-time streaming) and provided data for all days starting from day 1.

#### Signal Processing

LFPs recorded *via* RF streaming were imported and post-processed in MATLAB. The power spectral density (PSD) with a confidence interval of 95% of each 10-s time series was computed with the “pwelch” function using a rectangular window of 250 ms with 50% overlapping. The background neural activity was fitted between 4 and 40 Hz, as 1/f shaped noise, with the MATLAB function “robustfit.” Significant oscillatory activity was defined as the oscillatory activity whose power is above the neural background noise with a 95% confidence interval. A similar approach was used elsewhere, considering as true oscillations those being above 1/f noise of 0 or 0.5 standard deviation (Watrous et al., 2018; Goyal et al., 2021).

PSDs extracted from the embedded data were calculated as the average PSD in the ± 10 min interval around a clinical evaluation (Arlotti et al., 2018).

## Results

### Local Field Potential Recordings

Here, we report the results of LFP recordings in the first three patients implanted with the AlphaDBS System and involved in the pilot study NCT04681534. All patients were previously implanted with Medtronic 3389 electrodes, having four cylindrical contacts per side (left side: contacts 0-1-2-3, where contact 0 represents the most ventral and contact 3 represents the most dorsal; right side: contacts 8-9-10-11, where contact 8 represents the most ventral and contact 11 represents the most dorsal).

**FIGURE 3:**
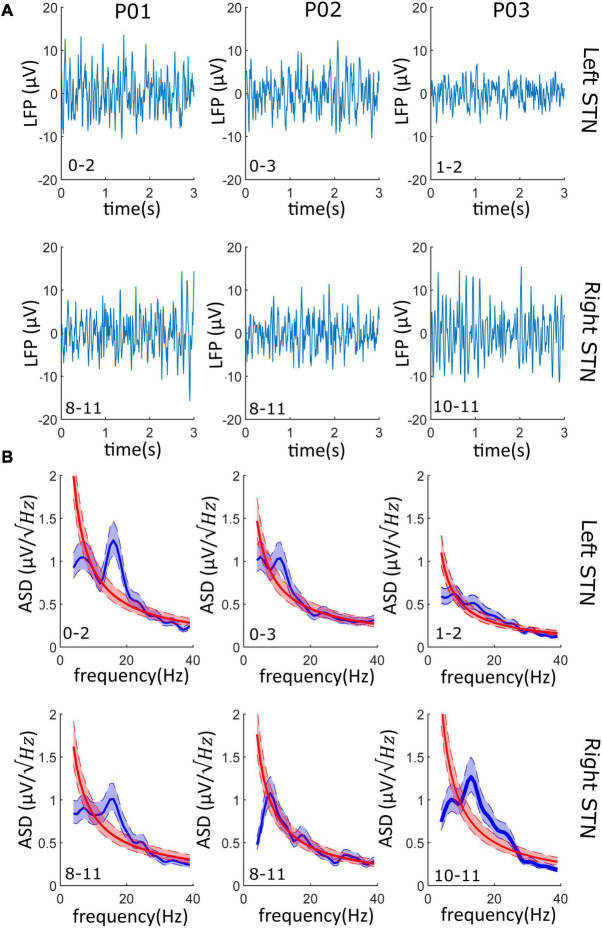
LFPs from the streaming mode: **(A)** Left and right LFPs time series. 3–s LFPs recordings are shown for both the left and right STN of each patient; on the bottom left corner, the contact pair used for recording is reported (i.e., “0–2” and “8–11”). **(B)** The PSD of the LFPs recordings of panel (a) is shown (blue line) and superimposed to the PSD of the 1/f background noise (red line). 95% confidence interval is shadowed around the PSD average (lighter blue and lighter red overlapped band). In at least one side per patient (four of the six recordings), the beta oscillations have a significative higher power than the background neural noise.

Data streamed showed limited data loss (average 2%) and no cardiac artifact in any recordings (Table 1). The signal-to-noise ratio (SNR) calculated on the beta peak was always greater than three logs, suggesting an optimal recording performance (Table 1). Representative raw LFPs and the correspondent PSDs obtained during data streaming are reported in Figure 3. As shown in Figure 3, significant (see METHODS – Signal Processing for explanation of “significant”) beta peak was found in at least one side per each patient. More specifically, the highest beta band activity was found in patients 01, 02, and 03 in contact pair 0–2 (left side), 0–3 (left side), and 10–11 (right side), respectively.

**TABLE 1:** Details of data streaming in all patients.

Patient	% of samples lost during streaming	SNR Ch1 (log)	SNR Ch2 (log)	EKG artifact observed
01	1.93%	4.22	4.13	0 of 6 tracks
02	1.92%	4.03	4.06	0 of 6 tracks
03	2.35%	3.83	4.17	0 of 6 tracks

*SNR, signal-to-noise ratio (log); EKG, electrokardiographic.*

**FIGURE 4:**
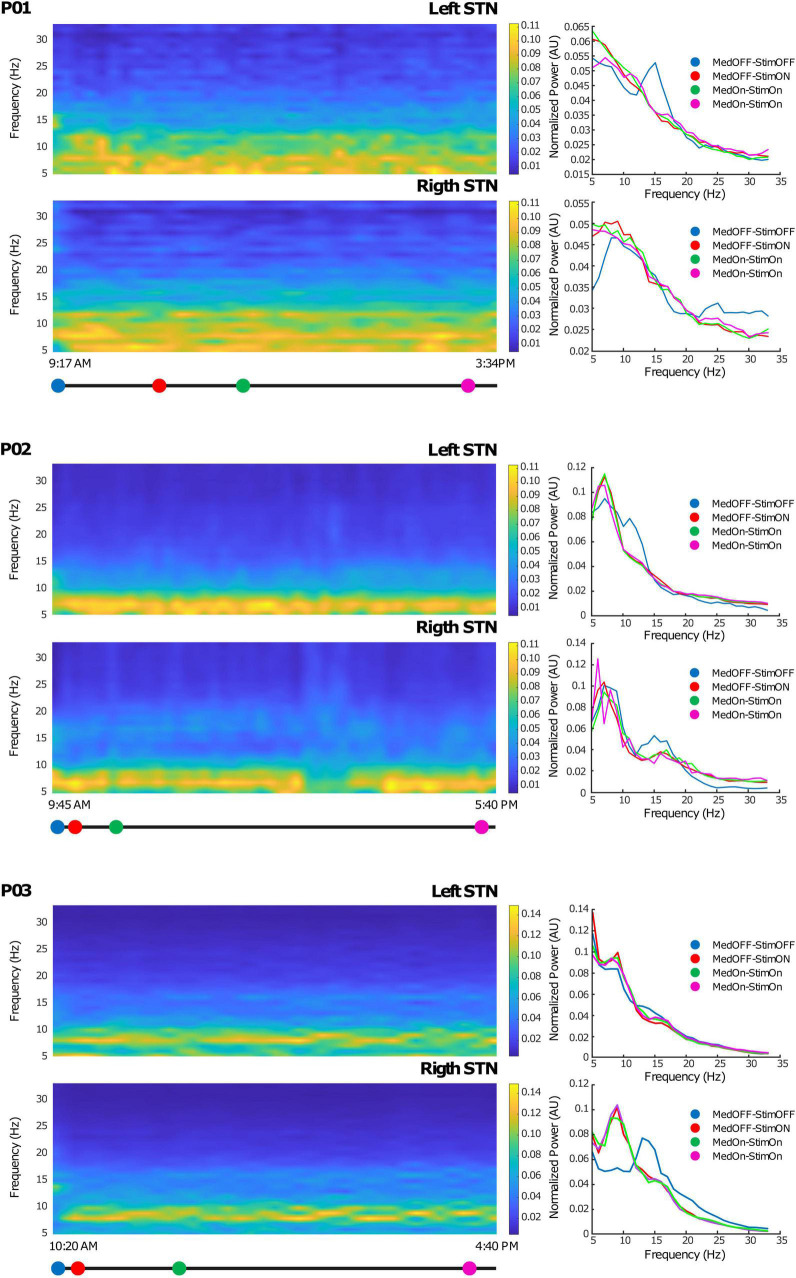
Chronic LFP recordings in the embedded mode: Left side: Time-frequency plots of six representative hours. The x-axis represents time and the y-axis frequency (from 5 to 35 Hz). The colored dots (blue, red, green, and magenta) correspond to clinical evaluations at MedOFF/StimOFF, MedOFF/StimON, MedON/StimON, and MedON/StimON, respectively. Right side: Amplitude spectrum of the LFPs of both the left and right STN extracted as the mean of the ± 10-min interval around the evaluation point (MedOFF/StimOFF, MedOFF/StimON, MedON/StimON, and MedON/StimON) obtained at the time indicated in the left side panels. Please note that, in MedOFF-StimOFF, a clear beta peak is present in the left STN of patients 01 and 02 and the right STN of patient 03. This beta peak was disappeared by the stimulation.

From the internal memory of the IPG, we downloaded the PSD of the contact pair selected from streamed LFPs, thus allowing a full monitoring of LFP fluctuation over time. The AlphaDBSipg stored the power spectrum every 10 min and downloaded it (together with the beta power value every minute and the stimulation value every 10 min) on the AlphaDBSpat at each recharging. Figure 4 shows the time-frequency spectra during 6 h of chronic recordings in the short-term follow-up conducted in clinic for each of the three patients. As shown, the embedded mode provided high-quality data that were successfully used for closed-loop stimulation. Note that, for patients 01 and 02, the recording configuration was symmetric (stimulation contact between recording contacts), whereas for patient 03, it was asymmetric (stimulation contact outside recording contacts). Iterative storing and downloading allowed for chronic data collection, providing highlights on the system functioning in terms of beta power tracking, closed-loop implementation, and neurophysiological monitoring.

### Stimulation Parameters

DBS parameter settings for all patients in cDBS mode are reported in Table 2. Despite the small number of patients, which prevents from running a statistical comparison, stimulation parameters were similar, for cDBS, using the AlphaDBSipg and the previously implanted IPG. The total electrical energy delivered per second (TEED) by AlphaDBSipg in cDBS was lower than the previous IPG in patient 01 (−116 μW), in patient 02 (−55 μW), and higher in patient 03 (+ 29 μW) (see Table 2).

**TABLE 2:** Parameter setting details.

			Previous IPG (cDBS mode)				AlphaDBSipg (cDBS mode)			
Patient	Stim Config	Imp (kΩ)	Amp (mA)	Freq (Hz)	PW (μs)	TEED** (μW)	Amp (mA)	Freq (Hz)	PW (μs)	TEED** (μW)
01	Left: C + 1−	0.84	4*	130	90	187	4	130	60	124
	Right: C + 9−	0.94	3.4*	130	90	143	3.5	130	60	90
02	Left: C + 1−	0.81	3.2*	130	60	80	2.4	130	60	45
	Right: C + 9−	0.86	2.4*	130	60	45	1.8	130	60	25
03	Left: C + 3−	1.47	2.5	130	60	49	3.0	130	60	70
	Right: C + 11−	0.96	2.3	130	60	41	2.5	130	60	49

*Previous implant voltage controlled. Current values were calculated using a simple translation to current on the basis of measured impedances parameter following Ohm’s law.

**TEED calculated using nominal 1 kΩ impedance.

## Discussion

Here, the AlphaDBS system presented and tested in patients implements a distributed architecture, allowing data collection and management for interfacing with the deep neural system and presents several innovative features that, combined altogether, create a reliable platform for aDBS and closed-loop neuromodulation applications.

First, the system provides fully artifact-free recordings, but, unlike other devices, it is stimulation agnostic, electrode configuration independent, and needless for back-end processing. In fact, we found that the system was capable to work both with different types of stimulation (different pulse widths in patient 01) and with asymmetric electrode configuration (as for patient 03). The stimulation artifact rejection has been achieved at the chip level (no blanking, no symmetrical sensing, and no back-end software) and not at the system level (Stanslaski et al., 2018). A further advantage of these features is that the IPG software is employed only for implementing the closed-loop strategy and not to mitigate the artifact, thus increasing the flexibility of the device for potential new closed-loop strategies that would not have to take care of artifact management.

The reliability of the sensing module is also demonstrated by the ability to provide a fully closed-loop aDBS in all patients: the performance of the sensing technology in rejecting the stimulation artifact allowed the implementation of the embedded linear proportional feedback aDBS, which is based on continuous sensing of beta band power, with consequent continuous adjustment of the stimulation amplitude in a proportional fashion (Arlotti et al., 2018; Guidetti et al., 2021), all done without any need of external processing. In addition, consistency between LFP features recorded through the AlphaDBS System and LFP features recorded in classical experimental settings was proved by the observation of a significant beta oscillatory activity detected in at least one contact pair for each patient and of a suppression of beta activity with concurrent reduction of the symptomatology during stimulation.

Second, data management has two major innovative features: (1) the capability to provide on-demand raw LFP streaming and (2) continuous embedded recording of a subset of data that are stored in the IPG and then downloaded to the patient controller at each recharging, without data loss or memory overwrite. These two, when combined, allows the system both to be used in experimental settings with high-fidelity real-time data, both when stimulation is OFF and ON, and also in clinical applications, collecting a significant amount of ecologic data with a download strategy that does not introduce additional burdens for the patient. In fact, because embedded data are downloaded while the patient is recharging and because the memory capacity was designed to fit the maximum time lapse allowed between two recharging sessions, data are never overwritten and are collected without the need of additional devices or intervention of the clinician. Therefore, data collection is also continuous when the patient is at home without any time constraint.

Finally, as expected, although the stimulation module requirements were designed on PD therapy, the DBS parameter space in dystonia is similar to that in PD (Magown et al., 2018): average stimulation voltage is around 3.3 V ± 0.6 V, average frequency is 131 Hz ± 5 Hz, and average pulse width ranges from 80 to 450 μs. Similarly, in essential tremor, DBS of the ventralis intermedium nucleus (Vim-DBS) can be successfully applied using the same ranges of parameters (Rodríguez Cruz et al., 2016). Therefore, the AlphaDBS System could be suitable also for dystonia and essential tremor. In all cases, the choice of specific parameters depends on the neurobiological electrical properties of the target neural populations (i.e., STN, GPi, and Vim), on the relative position between electrode and neurons’ ensembles, and on the expected mechanism of afferent/efferent neural structures inhibition/excitation.

The system has, however, some limitations. The AphaDBSipg has two sensing channels, cutting information at 40 Hz, thus introducing a limitation in the implementation of closed-loop algorithms based on gamma activity. However, the system architecture is modular and the sensing problem has been resolved at the chip level not at the design level (Stanslaski et al., 2018; Goyal et al., 2021), thus allowing sensing channel replacing by others with higher bandwidth including gamma (100 Hz cutoff frequency). Cutting information at 40 Hz is a selective and conservative choice for targeting beta power in PD applications while saving memory space and reducing the streaming load.

Similarly, the closed-loop algorithm implemented was conservatively chosen as a simple one, on the basis of previous experiences with external devices. It has several limitations largely debated in the scientific community (Beudel and Brown, 2016; Cagnan et al., 2019a,b; Krauss et al., 2021), especially related to the lack of relationship between beta activity and complex symptoms (e.g., gait/speech disturbances), and the limited time resolution of spectral features (Cagnan et al., 2019b). The effectiveness of the closed-loop algorithm, not only from a technical standpoint but also from a clinical endpoint, is the objective of the ongoing study. However, the AlphaDBS system closed-loop technology is firmware-controlled, and, because the sensing module does not require additional digital signal processing, all the capabilities of the microprocessor firmware can be used for closed-loop implementation.

In conclusion, the system here presented and tested can be considered as a proof of functioning of a clinically viable deep BCI for closed-loop stimulation delivery, reliable artifact-free recording, and chronic long-term data and neural signal management.

## Data Availability Statement

The raw data supporting the conclusions of this article will be made available by the authors, without undue reservation.

## Ethics Statement

The studies involving human participants were reviewed and approved by Comitato Etico Milano Area 2 (Milano) Comitato Etico Fondazione IRCCS Istituto Neurologico C. Besta (Milano) Comitato Etico Interaziendale A.O.U. Città della Salute e della Scienza di Torino—A.O. Ordine Mauriziano—A.S.L. Città di Torino (Torino) Comitato Etico per la Sperimentazione Clinica della Provincia di Padova (Padova) Bioethics Committee at the National Institute of Oncology of Maria Skłodowska-Curie (Warsaw) De Medisch Ethisch Toetsingscommissie van Maastricht UMC. The patients/participants provided their written informed consent to participate in this study.

## Author Contributions

MA, SM, and AP: system design and conceptualization. MA, MC, and AB: technology development. MA and SM: data analysis, clinical study design, and manuscript drafting. TM, EP, LB, PR, ML, RE, LR, SR, and MJ: study conduct. All authors: manuscript reviewing and approval.

## Conflict of Interest

MA and MC were employed by Newronika and held stock options. AB is a consultant for Newronika. AP, SM, and PR are founders and shareholders of Newronika. The study was funded by Newronika SpA. The funder had the following involvement with the study: study design of NCT04681534, signal collection and analysis (clinical data collection is performed by a CRO), the writing of this article, and the decision to submit it for publication. The remaining authors declare that the research was conducted in the absence of any commercial or financial relationships that could be construed as a potential conflict of interest.

## Publisher’s Note

All claims expressed in this article are solely those of the authors and do not necessarily represent those of their affiliated organizations, or those of the publisher, the editors and the reviewers. Any product that may be evaluated in this article, or claim that may be made by its manufacturer, is not guaranteed or endorsed by the publisher.
